# Understanding Dental Clinical Trainees’ Knowledge and Attitudes Toward Managing Diabetic Patients: Insights From a University Dental Training Center

**DOI:** 10.7759/cureus.75112

**Published:** 2024-12-04

**Authors:** Merin Mathew, Amal Alrayes, Maysam Al Blayhd, Shmoukh Alghumaiz, Sandhya A Methal, Rajeswari V

**Affiliations:** 1 Prosthetic Dental Sciences, Jouf University, Sakaka, SAU; 2 Dentistry, Jouf University, Sakaka, SAU; 3 Physiology and Biochemistry, Bharati Vidyapeeth (Deemed to be University) Dental College and Hospital, Navi Mumbai, IND; 4 Microbiology, Viswabharathi Medical College and General Hospital, Kurnool, IND

**Keywords:** attitude, dental education, diabetes mellitus, knowledge, oral manifestations, questionnaire

## Abstract

Background: Dental patients with diabetes require comprehensive care to maintain their metabolic stability during dental treatments. Clinicians' understanding of diabetes symptoms and management strategies is essential for minimizing risks associated with dental procedures, especially since undiagnosed diabetic patients may also seek dental care. This study aimed to assess dental clinical trainees' knowledge and attitudes toward managing diabetic patients.

Materials and methods: A quantitative study was conducted using a custom-made, pre-tested, self-reported survey. The independent variables included the age, gender, and educational level of the participants. The primary outcomes were evaluated by analyzing participants' knowledge and attitudes regarding dental conditions related to diabetes and the management of diabetic patients in dental clinics.

Results: Data were analyzed using Spearman’s, Mann-Whitney, and Kruskal-Wallis tests. A significant positive correlation was observed between participants' knowledge and attitudes toward diabetic patient management (p<0.01). The educational level of participants significantly impacted their knowledge scores (p<0.05). Additionally, both gender and study level influenced attitude scores (p<0.001).

Conclusion: The findings highlight the importance of educational programs designed to raise awareness and enhance dental students' competence in managing diabetic patients. Such training is crucial for ensuring dental professionals are well-prepared to provide effective care for diabetic individuals.

## Introduction

Diabetes mellitus (DM) is a chronic endocrine disorder caused by insulin deficiency or resistance. Insulin is a hormone produced by β-cells in the pancreas that helps maintain normal blood glucose levels [1-2]. A large portion of the world's population is affected by diabetes and its associated complications. Dental problems should not be ignored in patients with DM. Poor oral health can be a major barrier to maintaining normal blood glucose levels in diabetic patients. DM is a metabolic disorder with long-term complications, often accompanied by comorbidities such as obesity, hypertension, cardiovascular problems, and renal diseases. Emergencies such as cardiac failure, cerebrovascular disease, pneumonia, and infections are common among diabetic patients and can be fatal if left untreated. Dental practitioners can assist in diagnosing DM based on early oral symptoms. Preventative measures to control metabolic health and maintain oral health pave the way for a healthier lifestyle. Therefore, dental practitioners play a significant role in improving the quality of life for diabetic patients [1-4].

According to a World Health Organization (WHO) report, more than 400 million people are affected by this chronic disease, with global prevalence increasing each year. Diabetes has become a major cause of blindness, kidney failure, heart attacks, stroke, and lower limb amputation. In 2016, it was identified as the seventh leading cause of death by the WHO [5]. Consequently, dental patients with DM require comprehensive care to avoid disruptions in metabolic homeostasis. Both diagnosed and undiagnosed diabetic patients may seek various dental treatments. Proper precautions are essential while treating diabetic patients to avoid life-threatening risks [1,6-9]. Therefore, clinicians must have a thorough understanding of the symptoms and management strategies for diabetes to minimize the risks associated with dental treatment [10-12]. Additionally, patients need to be educated on diabetes-related oral health issues and the importance of maintaining oral hygiene through preventative care routines to minimize dental complications [8,9].

A thorough understanding of diabetes and proper management of diabetic patients in dental clinics is crucial. Hence, this study aims to evaluate the knowledge and attitudes of Jouf University’s dental students and interns regarding diabetic patient management in dental clinics.

## Materials and methods

Study design and setting

A quantitative study was conducted at the College of Dentistry, Jouf University, involving dental students from the third to fifth year and interns working in the clinical setting. A self-reported survey was used to assess the knowledge and attitudes of these participants regarding the management of diabetic patients in dental clinics. The survey instrument was developed based on validated tools and relevant literature in diabetes management within dental settings [4-13] to ensure content validity. Expert input from faculty members specializing in diabetes management and dental education was incorporated to finalize the survey questions. To refine the instrument further, a pilot test was conducted with a small subset of dental students and interns.

Data collection took place between 15 March 2022 and 02 May 2022, with participants who voluntarily provided informed consent included in the study. Those who did not consent failed to complete the questionnaire, or did not provide basic demographic information were excluded. To minimize potential biases, anonymity and confidentiality were assured during the consent-taking process, encouraging honest and accurate responses.

Sample size calculation

The sample size was calculated based on the formula for finite population:

\[ n = \frac{NX}{X + (N - 1)} \]

\[ X = \frac{\left(\frac{Z_{\alpha/2}}{2}\right)^2 p(1-p)}{(MOE)^2} \]

Also, Zα/2 is the critical value of the normal distribution at α/2 (for a confidence level of 95%, α is 0.05, and the critical value is 1.96), MOE is the margin of error (5%), and p is the sample proportion (50%).

No published study on this topic was identified from the extensive review of the literature in the KSA, covering the entire healthcare system. Therefore, the research team took the expected proportion (p) as 50% to get the maximum sample size. N is the population size, which is the total number of students and interns in the Jouf University dental training center = 160.

Therefore,

\[ X = \frac{(1.96)^2 (0.5)(1 - 0.5)}{(0.05)^2} = 385.6 \]

\[ n = \frac{(160)(385.6)}{544} = 113 \]

Data collection

Data collection for this study was carried out using a customized and pretested questionnaire. The study comprised two main sections. The first section gathered demographic data, including age, gender, and educational level, from participants, while the second section focused on assessing their knowledge and attitudes through a structured set of questions. This included queries about their prior participation in educational programs related to the management of diabetic patients in dental clinics.

Participants were approached in person within the clinical setting of the College of Dentistry, Jouf University. Each participant received a detailed informed consent form that outlined the purpose, objectives, and significance of the study, emphasizing the importance of understanding and improving the management of diabetic patients in dental care. Participants were informed that their role would involve completing a questionnaire based on their experiences, attitudes, and knowledge, with no additional follow-up commitments. Participation was entirely voluntary.

The informed consent form assured participants that no identifying information would be collected or disclosed. Personal data, including names or other personal details, were neither requested nor recorded. All data collected would remain anonymous and confidential, with no identifying information being published or shared. Participants were explicitly instructed not to write their names or any identifying information on the questionnaire.

The questionnaires were distributed in person in hardcopy format. After completion, male and female students from each academic year were asked to submit their completed forms to the designated leader of their respective level. The completed forms were then collected and submitted by the group leader. This method ensured an organized collection process while maintaining anonymity and preserving the integrity of participant responses. All responses were handled confidentially to protect the privacy of all participants. A shared Google spreadsheet (Google LLC, Mountain View, CA, USA) was then used to simplify the process of data entry and collaboration among researchers. To ensure data integrity and minimize the risk of unauthorized edits, each section of the spreadsheet was restricted to specific researchers assigned to that section. Permissions were managed to allow editing only by the designated researcher, while other team members had view-only access to those sections. This approach ensured data accuracy and accountability throughout the data management process.

Ethical consideration

The Local Committee on Bioethics approved the study design and data collection at Jouf University (approval number: 3-05-43) on 10 March 2022. All participants were aware of the study objectives to ensure complete privacy and confidentiality. In addition, participants were advised that they could withdraw from the study at any time without consequences.

Statistical analysis

The Microsoft Excel data (Microsoft Corporation, Redmond, WA, USA) collected from the Google spreadsheet was exported to SPSS Statistics version 21 (IBM Corp. Released 2012. IBM SPSS Statistics for Windows, Version 21.0. Armonk, NY: IBM Corp.) and encoded. Initially, a Wilk-Shapiro test was performed to assess the normality assumption of the collected data. A skewed distribution was observed, and non-parametric tests, Spearman's test, Mann-Whitney test, and Kruskal-Wallis test were performed based on the independent variables considered for the study. A p-value of <0.05 was considered statistically significant.

## Results

Demographic characteristics of the participants

**Figure 1:**
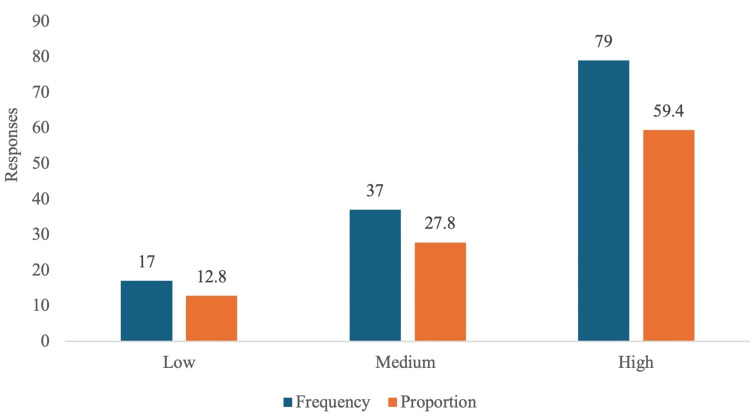
Knowledge categories

**Figure 2:**
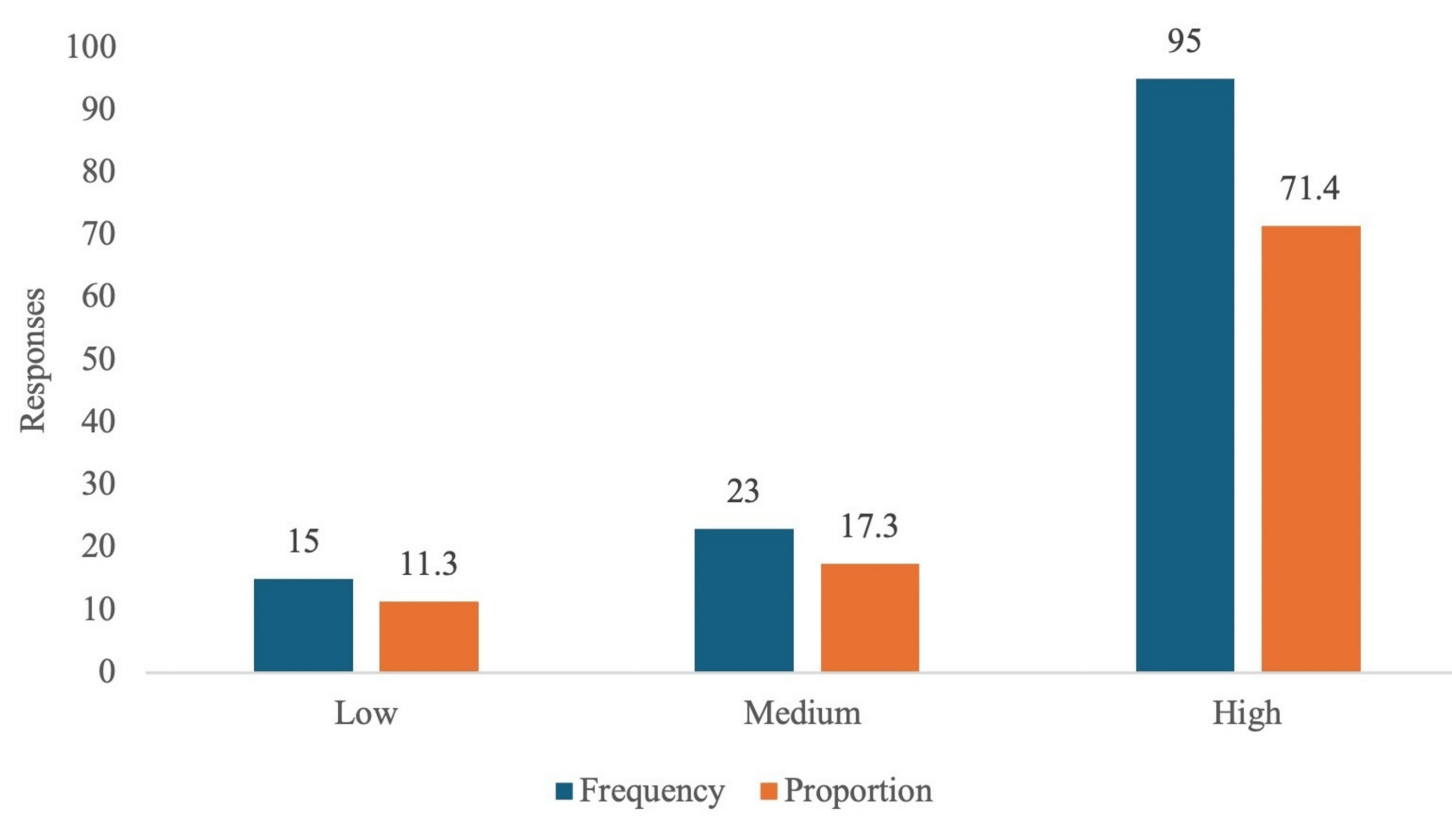
Attitude categories

Based on the population (p=160) considered, 113 participants' responses were sufficient to validate the study. However, we received 133 responses, all of which met the inclusion criteria and were included for evaluation. Table 1 describes the demographic data of the participants, while Figure 1 and Figure 2 illustrate the knowledge and attitude categories in this study.

**Table 1:** Background details SD: standard deviation, BDS: Bachelor of Dental Surgery

Variables	Frequency (n)		%
Age (mean ± SD) = 23 ± 1			
Gender			
Male	77	57.9	
Female	56	42.1	
Level of study			
III BDS	36	27.1	
IV BDS	31	23.3	
V BDS	34	25.6	
Interns	32	24.1	

Association between background details and knowledge score

The age and gender of the participants did not influence the knowledge score. However, the study level of the participants showed a significant difference. Fifth-year dental students exhibited maximum knowledge scores (Table 2).

**Table 2:** Association between background details and knowledge score * Spearman's test, ** Mann-Whitney test, *** Kruskal-Wallis test, bold: statistically significant (p<0.05) BDS: Bachelor of Dental Surgery

Variables	Mean rank/rho value	p-value
Age*	0.025	0.777
Gender**		
Male	69.95	0.292
Female	62.94	
Level of study***		
III BDS	63.63	0.007
IV BDS	60.53	
V BDS	85.99	
Interns	56.89	

Association between background details and attitude score

The attitude scores differed significantly based on the gender and study level of the participants. Male participants exhibited better attitudes compared to female participants. Similarly, a comparable trend in knowledge scores was observed for attitudes regarding the variable of study level. The maximum positive attitude was observed among fifth-year dental students (Table 3).

**Table 3:** Association between background details and attitude score * Spearman's test, ** Mann-Whitney test, *** Kruskal-Wallis test, bold: statistically significant (p<0.05) BDS: Bachelor of Dental Surgery

Variables	Mean rank/rho value	p-value
Age*	0.074	0.394
Gender**		
Male	72.99	0.025
Female	58.77	
Level of study***		
III BDS	74.25	0.000
IV BDS	65.74	
V BDS	81.38	
Interns	44.78	

Analysis between knowledge and attitude toward diabetic patient management

A significant positive correlation was observed between the knowledge and attitude of the dental students (Table 4). As knowledge increased, attitudes also improved. The open-ended inquiries revealed participants' knowledge and attitudes toward diabetic patient management techniques. Most participants suggested scheduling early appointments, while a few recommended providing proper counseling before treatment to alleviate diabetic patients' anxiety about dental procedures. Additionally, participants were aware of the possible complications associated with diabetic patients during dental treatment, including the risks of syncope, hypoglycemia, and hyperglycemia. However, most considered delayed healing to be an immediate complication during dental treatment.

**Table 4:** Spearman’s correlation analysis between knowledge, attitude, and practice toward diabetic patient management * Statistically significant at 0.01 (two tailed)

	Attitude	
Knowledge	rho value	p-value
	0.511	<0.001*

Most importantly, many students were unaware of how to manage medical emergencies involving diabetic patients during dental treatment. However, those who had previously attended educational programs on diabetic patient management provided appropriate responses. They suggested stopping dental treatment if symptoms of hypoglycaemic shock occurred. They also recommended checking the blood glucose level and administering fast-acting carbohydrates if necessary to prevent further complications. If the patient was unconscious or had difficulty swallowing or breathing, they advised seeking immediate medical attention and administering subcutaneous glucagon.

## Discussion

Oral health problems, such as periodontal diseases, dental caries, and xerostomia, are common in people with uncontrolled or poorly controlled diabetes. Diabetes and periodontal disease have a bidirectional relationship: periodontal disease can worsen glycemic control, and poorly managed diabetes can lead to periodontitis. Additionally, uncontrolled diabetes and certain medications used for diabetes management can cause xerostomia by impairing the normal function of the salivary glands. Elevated blood glucose levels also increase glucose in the saliva, creating an environment that promotes microbial accumulation and biofilm formation on the tooth surface, thereby increasing the risk of dental caries and gum disease. Therefore, it is essential to educate diabetic patients on maintaining oral hygiene. Dental practitioners should have a thorough understanding of diabetes-related oral health problems and risk factors [6-11]. A study conducted among future dentists at Jouf University, Saudi Arabia, observed a positive correlation between knowledge and attitude toward diabetic patient management. Similar results were found in studies among oral health professionals in Australia and India [12,13].

As shown in Table 1, participants' age, gender, and level of education were considered to assess their knowledge and attitudes toward diabetic patient management in dental clinics. Age and gender differences did not affect participants' knowledge. However, academic level showed a significant variation in the knowledge score, with interns having lower knowledge compared to other study groups (Table 2). This may be due to the structure of the dental curriculum, where medical courses are integrated in the initial years to build a strong foundation for understanding diabetes and its systemic implications. Additionally, problem-based learning sessions are incorporated to enhance the application of knowledge in clinical contexts. As students progress, particularly in the later years, the focus shifts toward dental specialties, which may reduce the emphasis on medical subjects. In the fifth year, the "Problem Solving in Dentistry" course specifically addresses the management of medically compromised patients, reinforcing both practical and theoretical knowledge relevant to dental practice. These findings align with those of Komerik et al., who reported that dental students showed negative enthusiasm toward learning medical subjects in their curriculum. The dental students in their study felt that certain course content, including aspects of the human body unrelated to dentistry, was unnecessary and that they quickly forgot this information [14].

**Figure 3:**
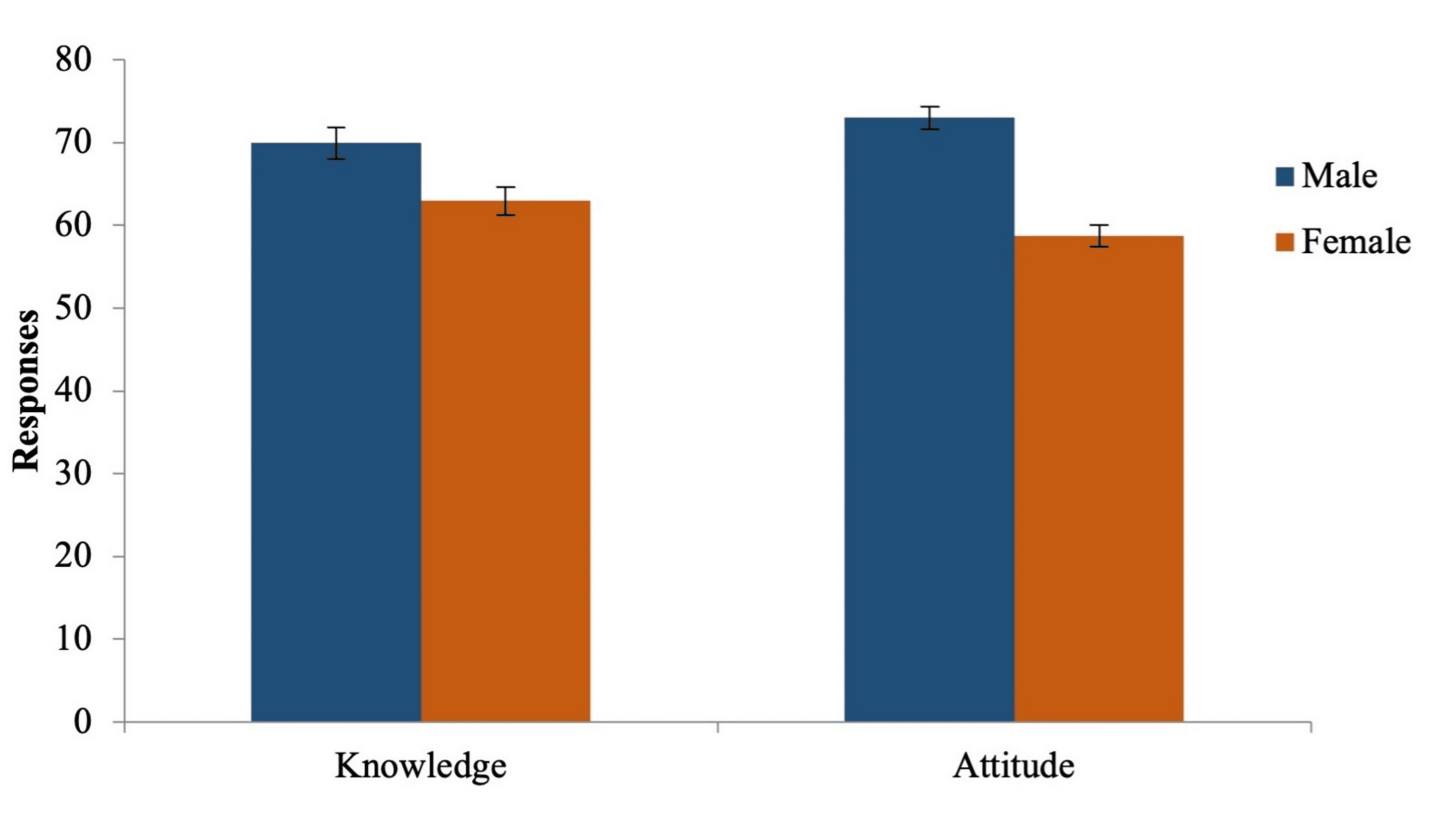
Knowledge and attitude of the participants based on gender

**Figure 4:**
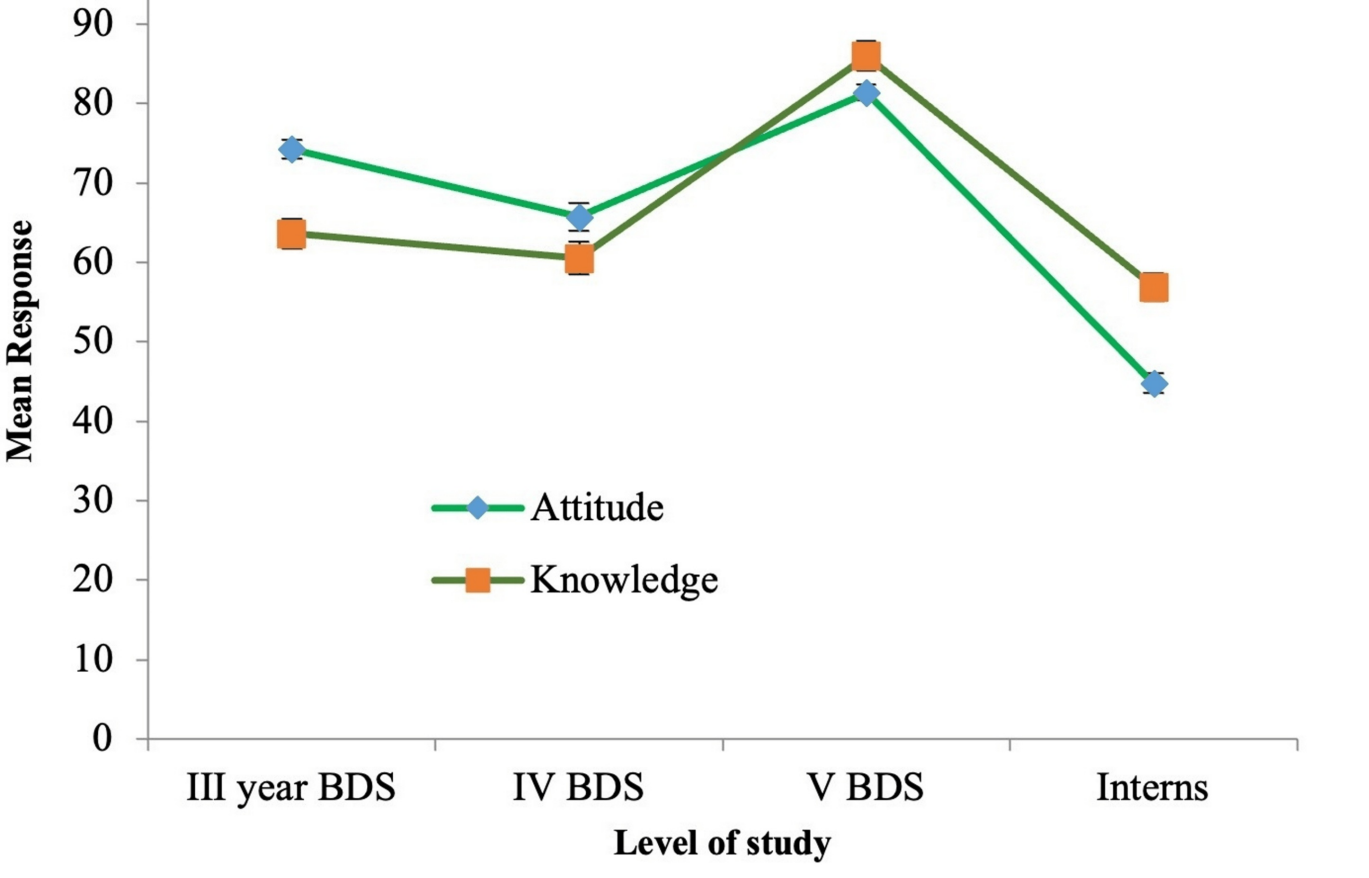
Knowledge and attitude of the participants based on the level of study BDS: Bachelor of Dental Surgery

Furthermore, attitudes toward managing diabetic dental patients varied based on gender and academic level. As shown in Figure 3, males displayed more positive attitudes than females despite there being no significant difference in knowledge. This difference may be attributed to higher levels of confidence in managing diabetic patients, which could result from prior exposure to and participation in workshops specifically focused on diabetic patient management. These workshops likely provided them with a stronger foundation of both knowledge and practical experience, enhancing their comfort levels and promoting a more proactive approach to patient care. A similar trend in knowledge scores was observed for attitudes based on academic level (Figure 4). The least positive attitude was observed among interns (Table 3), which is consistent with Mussalo et al.'s findings. They reported that students' interest in biomedical sciences declined as they progressed through their dental program and entered the clinical phase [15]. The current study supports these findings, confirming that positive attitudes toward managing patients in clinical settings are closely linked to both knowledge and confidence levels [6-12].

The responses related to participants' knowledge and attitudes highlighted the need for educational programs on diabetic patient management for dental students. Although some participants demonstrated basic knowledge of diabetes, most were unaware of the proper management of diabetic emergencies during dental treatment. Clinicians must be efficient in managing hypoglycemic shock, which is associated with symptoms such as confusion, shakiness, tremors, agitation, anxiety, sweating, dizziness, tachycardia, a feeling of impending doom, seizures, and loss of consciousness [16-20]. If any of these symptoms arise, it is advisable to stop dental treatment immediately. Administering 15 grams of rapid-acting carbohydrates is recommended to prevent further complications. Additionally, monitoring glucose levels is essential to determine if the patient requires further treatment. If the patient is unconscious or has difficulty swallowing or breathing, immediate medical attention should be sought, and glucagon should be administered subcutaneously [16-25]. The participants in the current study were not confident in recognizing hypoglycemic shock symptoms and indicated that they would seek advice from supervisors, reflecting their anxiety about managing such patients in the dental clinic. However, students who had attended a previous workshop on diabetic patient management showed a more positive attitude toward managing medical emergencies.

The participants in this study represent the future of dental practice. Therefore, the findings suggest that special training in diabetic patient management should be provided to dental students during their internship years. To build on these findings, it is recommended to integrate hands-on workshops, role-playing scenarios, and practical resources such as clinical posters or digital guides into the dental curriculum to strengthen the application of skills. Additionally, conducting regular evaluations, including periodic surveys, can help assess retention and ensure the ongoing effectiveness of the training. This would help dental graduates recall the basic medical knowledge learned in the early years of their graduate programs and apply that knowledge in practice.

Limitations of the study

The study's findings may not be generalizable beyond Jouf University due to differences in educational structures. Participants' knowledge and attitudes were potentially influenced by recall bias and varied prior training experiences. Specifically, differences in prior educational opportunities were evident, as some students attended workshops on diabetic patient management, potentially skewing the results toward more positive attitudes among those with additional exposure. Furthermore, a study involving a larger, more diverse population is recommended to enhance the generalisability of the findings.

## Conclusions

The study found that education level significantly influenced dental students' knowledge and attitudes toward managing diabetic patients, with fifth-year students performing better than interns. Gender differences impacted attitudes, though knowledge remained unaffected, while age showed no influence on either. A positive correlation between knowledge and attitude was observed, particularly among students with prior training. These findings highlight the need for specialized training, interactive workshops, regular assessments, and resources like posters to enhance students' confidence and preparedness in managing diabetic patients.

## Appendices

Understanding dental clinical trainees’ knowledge and attitudes toward managing diabetic patients: Insights from a University Dental Training Center

Dear participant,

Please mark your response to the following questions:

*Part I*

1.     Age……………. Years

2.     Gender: Male ------                    Female ----------

3.     You are a …… (tick one of the following)

                                    Intern---------

                                    5th academic year student---------

                                    4th academic year student --------

                                    3rd academic year student ---------

Part II

**Table 5:** Part II questions

	Knowledge-based questions	Yes	No
1	Diabetic mellitus (DM) can go undiagnosed for many years after it actually begins.		
2	You are aware that individuals with both diagnosed and undiagnosed DM may approach dental treatment.		
3	When a diabetic patient comes to you, it is necessary to take precautions while scheduling an appointment for him/her.		
4	Is it important to know the medications your diabetic patient might be taking		
5	A consent letter from the doctor/ physician/ diabetologist before starting any dental procedure is required.		
6	It is important to have a device to monitor blood glucose levels in your clinic.		
7	A glycated hemoglobin level (HBA1c) of less than 5.7% is indicative of good glycaemic control.		
8	You need to counsel the patients regarding the usage of oral hygiene aids.		
9	Antibiotic coverage before commencing any surgical procedure on a diabetic patient is important.		
10	Treatment of periodontal disease by scaling and root surface debridement may improve glycaemic control in people with DM.		
	Attitude-based questions		
11	Dental practitioners’ knowledge of a patient’s overall health is important for achieving optimal oral health outcomes.		
12	Screening patients for DM risk in the dental setting could offer new opportunities to identify patients with possible undiagnosed DM or pre-DM.		
13	Screening for DM in the dental setting will help patients understand the link between uncontrolled DM and poor periodontal health.		
14	It is appropriate for dentists to screen patients for DM in the dental setting.		
15	It is important for dentists to perform or conduct chair-side screening for type 2 DM.		
16	You feel comfortable providing oral healthcare to patients with DM.		
17	Periodontal screening and subsequent follow-up can facilitate conversations with physicians when patients seek their treatment.		
18	Have you attended any educational programs specifically tailored for dental students on the management of diabetic patients?		

Open-Ended Questions

What special measures will you take for controlling anxiety in a diabetic patient?

------------------------------------------------------------------------------------------------------------------------------------------------------------------------------------------------------------------------------------------------------------------------------------------------------------------------------------

What are the complications associated with dental treatment of uncontrolled diabetic patients?

------------------------------------------------------------------------------------------------------------------------------------------------------------------------------------------------------------------------------------------------------------------------------------------------------------------------------------

What are the major medical emergencies encountered while treating a diabetic patient? How will you manage it?

------------------------------------------------------------------------------------------------------------------------------------------------------------------------------------------------------------------------------------------------------------------------------------------------------------------------------------------------------------------------------------------------------------------------------------------------

Thank you for your participation!
